# The relationship among socioeconomic status, social support and frailty: is there a gender difference?

**DOI:** 10.1007/s40520-025-03013-8

**Published:** 2025-04-02

**Authors:** Ping Dong, Xian-qi Zhang, Wen-qiang Yin, Zi-yuan Li, Xiao-na Li, Min Gao, Yong-li Shi, Hong-wei Guo, Zhong-ming Chen

**Affiliations:** 1 School of Management, Shandong Second Medical University, Weifang, Shandong China; 2 School of Public Health, Shandong Second Medical University, Weifang, Shandong China

**Keywords:** Socioeconomic status, Social support, Frailty, Gender difference, Older adults, Rurality

## Abstract

**Objective:**

This study aimed to determine the relationship among socioeconomic status, social support and frailty, and its gender difference.

**Methods:**

Education and income were combined to indicate the socioeconomic status. The Social Support Rating Scale (SSRS) was used to measure the level of social support. Frailty was measured by the FRAIL Scale. Mediation effects were analyzed using the PROCESS 4.1 macro in SPSS version 26.0.

**Results:**

Among the 936 participants, socioeconomic status had a direct effect on frailty (effect = − 0.088, 95% *CI*: − 0.142, − 0.021). Social support was an indirect pathway for the relationship between socioeconomic status and frailty (effect = − 0.011, 95% *CI*: − 0.023, − 0.003), accounting for 11.11% of the total effect. Stratified by gender, we found that the total, direct and indirect effects of socioeconomic status on frailty were significant only in the female subsample.

**Conclusion:**

Overall, there was a significant association between socioeconomic status and frailty among the rural older adults, and social support mediated this relationship. However, there were gender differences in the association among socioeconomic status, social support and frailty. Specifically, the correlation between socioeconomic status and frailty and the mediating role of social support were found only in the female subsample. The public health sector should focus on the rural older adults with low socioeconomic status and lack of social support, taking targeted interventions to avoid and delay the occurrence and progress of frailty.

## Introduction

At present, population aging has become an important public health problem in the world. It is projected that by 2050, the global population over the age of 65 will exceed 2 billion [1]. In recent years, the elderly population in China has been growing, accounting for 21.1% of the total [2], entering the accelerated stage of aging. Frailty is considered to be the most prominent health problem in the context of population aging [1]. It refers to the decreased function of multiple physiological systems of the human body, resulting in the increased susceptibility to stressors [3]. A recent study reported the prevalence of frailty among older adults in China reached 37.6% [4], and the prevalence was higher in rural areas than in urban areas [5]. Frailty is a predictor of many adverse health outcomes. It can not only increase the risk of physical health impairment, such as dementia, falls and death [6–8], but also threaten an individual’s mental health, leading to loneliness, anxiety and depression [9–11]. At the same time, the burden of medical services will increase [12, 13]. Studies have shown that frailty was reversible, and the reversal rate was significantly higher in prefrailty than in frailty [14]. Prefrailty is the transition from health to frailty, which is more prevalent but poses less threat to the health of older adults [15]. Therefore, the early identification and intervention of frailty can be beneficial in avoiding and delaying the occurrence and progress of frailty, as well as facilitating the transition of frail and prefrail individuals to the healthy state.

Socioeconomic status is a comprehensive indicator with multiple dimensions, including education, income and occupation [16, 17]. The influence of socioeconomic status on an individual’s health extends throughout his or her life cycle [18]. People with higher socioeconomic status are generally in better health [19]. For some patients, socioeconomic status also affects their quality of life and health by influencing the effective use of health resources and services [20]. Previous studies have shown that frailty was more likely to occur in groups with low socioeconomic status [21]. Higher levels of education and income can reduce the risk of frailty [22]. Although existing research has demonstrated the relationship between socioeconomic status and frailty. However, it is not clear which underlying mechanisms may contribute to greater frailty in older people with lower socioeconomic status, which requires further exploration.

As a key factor of active aging [23], social support is defined as the emotional, informational, and instrumental help that individuals can obtain from their own social network [24]. Good social support can buffer the stimulation of external pressure and play an important role in improving self-efficacy and promoting physical and mental health [25, 26]. Several studies have found a positive association between social support and socioeconomic status [27, 28]. Results from a Canadian survey suggested that high socioeconomic status may imply high levels of social support [29]. In addition, a longitudinal study involving middle-aged and elderly people in the Ruhr region of Germany by Nico Vonneilich et al. found that individuals with high socioeconomic status generally had more positive social relationships [30], whose functional aspect is exactly social support [31, 32]. Similar to socioeconomic status, frailty has also been reported to be significantly associated with social support [33, 34]. A 10-year epidemiological survey conducted in China discovered that the risk of frailty decreased with increasing levels of social support [35]. A study performed in Thailand came to a similar conclusion that inadequate social support increased the probability of frailty and prefrailty [36]. The clinical practice guidelines developed by the working group of the International Conference on Fragility and Sarcopenia Research (ICFSR) also mentioned that social support should be provided to all older adults with frailty according to need [37].

In conclusion, although a large body of evidence has proven that both socioeconomic status and social support have a direct impact on frailty, the specific potential mechanisms among the three have not yet been clarified. At the same time, previous studies have shown that there are gender differences in socioeconomic status [38]. Compared to males, inequality in socioeconomic status is higher and more strongly related with health status among females [39]. In addition, there is also a disparity in the size of social networks of males and females [40]. Spouses are generally the main social relationship that males rely on [41]. The difference is that, in addition to their spouses, females rely more than males on other relationships for social support, thus having larger social networks [42]. This may lead to more social support being accessed and utilized by females, resulting in a certain degree of gender difference in the link between social support and frailty. Currently, there is still a lack of effective measures to prevent and delay frailty in older adults. Understanding the relationship between socioeconomic status and frailty and its underlying mechanisms is of great value to help identify individuals at greater risk of frailty. Therefore, our study aimed to (1) investigate the relationship between socioeconomic status and frailty among rural older adults; (2) determine the mediating role of social support in the above relationship; (3) examine whether the relationship among socioeconomic status, social support and frailty differs by gender.

## Methods

### Study design and sample

The present study, was a cross-sectional survey conducted from September to December in 2023 in an eastern province of China. The details of the research program have been described in other articles [43]. In short, a certain number of residents from 216 villages or communities were selected for a questionnaire survey to identify our potential subjects. The eligible population included rural residents aged ≥ 60 years who volunteered to participate in the study. We excluded older adults with severe physical and / or cognitive impairments. In addition, older adults with missing data on key variables in the questionnaire were also not included. The study complied with the principles of the Declaration of Helsinki and was approved by the Medical Ethics Committee of Weifang Medical University (2021YX-066). All participants were provided written informed consent before the survey. Finally, 936 participants were eligible for inclusion in this study.

### Assessment of frailty

The FRAIL Scale was used to assess how frail the participants were [44]. This scale is a 5-point test that includes tests for fatigue, resistance, ambulation, illness and loss of weight. The higher the score, the more serious the frailty [45].

### Assessment of socioeconomic status

With reference to previous studies [46–48], socioeconomic status is usually combined by three indicators: education, occupation and income. However, this study focused on the rural older adults, most of whom were in non-working state and engaged in agricultural production activities before retirement. Occupation was not a highly heterogeneous indicator. Therefore, it was not included. We used annual household income per person and educational attainment to evaluate participants’ socioeconomic status. Quartiles were applied to divide annual household income per person (RMB) into four categories: < 2000 yuan = 1, 2000 − 4999 yuan = 2, 5000 − 9999 yuan = 3, ≥ 10,000 yuan = 4. We created three categories of educational attainment: primary and below = 1, middle school = 2, and high school and above = 3. The scores of the two indicators were added together to obtain the score of socioeconomic status, which ranges from 2 to 7. A high total score indicated high socioeconomic status.

### Assessment of social support

Social support was measured based on the Social Support Scale (SSRS). The scale includes three dimensions: the objective and subjective support received and the utilization of the above support [49]. The total score is 66. The higher the score, the higher the level of social support [50].

### Assessment of covariates

Covariates included gender (male, female), age (60–69 years, 70–79 years, ≥ 80 years), marital status (currently married, others), chronic disease (none, have), body mass index (BMI) and sleep quality. BMI is a commonly applied indicator for screening and diagnosing obesity, which is calculated by dividing weight (kg) by height squared (m^2^) [51]. The Pittsburgh Sleep Quality Index Scale (PSQI) was employed to estimate the sleep quality of subjects during the latest month. Scores on this scale range from 0 to 21. The lower the score, the better the sleep quality [52].

### Statistical analysis

First, we described the basic information of the total sample, the male subsample and the female subsample, respectively. Frequencies (n) and percentages (%) were used to represent categorical variables. Numerical variables were denoted as *M* (*P*_25_, *P*_75_) since they did not fit the normal distribution. Second, the associations among the main variables were examined by Spearman’s correlation analysis. Finally, controlling for all covariates, Model 4 of the PROCESS 4.1 macro was used for mediation analysis: (1) linear regression analysis was performed to explore the association between socioeconomic status and frailty, (2) linear regression analysis was used to test the association between socioeconomic status and social support, and (3) the linear regression analysis was used to further explore the relationship between socioeconomic status and frailty when social support was included as a mediator. The bootstrap method based on 5000 samples was applied to test the total, direct and indirect effects. The significance of results was determined by not including 0 between the upper and lower limits of the 95% *CI*. All analyses were performed using SPSS version 26.0. Statistical significance was set at *P* < 0.05 (2-tailed).

## Results

### Characteristics of the participants

As shown in Table 1, the participants consisted of 359 males and 577 females. 91.7% were aged 60–79 years. 81.4% were currently married. 88.6% had at least one chronic disease. The median BMI and sleep quality were 24.97 and 7.00, respectively. The median socioeconomic status of males and females was equal at 4.00. However, compared to males, females had lower levels of social support (median = 41.00), more severe frailty (median = 1.00).

**Table 1:** Participant characteristics

Variables	Total (*n* = 936)	Males (*n* = 359)	Females (*n* = 577)
Age (years), n (%)			
60 − 69	427 (45.6)	168 (46.8)	259 (44.9)
70 − 79	431 (46.1)	157 (43.7)	274 (47.5)
≥ 80	78 (8.3)	34 (9.5)	44 (7.6)
Marital status, n (%)			
Currently married	762 (81.4)	308 (85.8)	454 (78.7)
Others^a^	174 (18.6)	51 (14.2)	123 (21.3)
Chronic disease, n (%)			
None	107 (11.4)	50 (13.9)	57 (9.9)
Have	829 (88.6)	309 (86.1)	520 (90.1)
BMI, *M* (*P*_25_, *P*_75_)	24.97 (22.86, 27.34)	24.68 (22.86, 26.95)	25.39 (22.89, 27.34)
Sleep quality, *M* (*P*_25_, *P*_75_)	7.00 (5.00, 10.00)	7.00 (5.00, 10.00)	7.00 (5.00, 9.00)
Socioeconomic status, *M* (*P*_25_, *P*_75_)	4.00 (3.00, 5.00)	4.00 (3.00, 5.00)	4.00 (3.00, 5.00)
Social support, *M* (*P*_25_, *P*_75_)	42.00 (37.00, 46.00)	42.00 (38.00, 47.00)	41.00 (37.00, 46.00)
Frailty, *M* (*P*_25_, *P*_75_)	1.00 (0.00, 2.00)	0.00 (0.00, 2.00)	1.00 (0.00, 3.00)

^a^ Others includes single, divorced and widowed

### Correlation between study variables

Table 2 shows the correlation between socioeconomic status, social support, and frailty. We found that in the total sample, socioeconomic status was positively associated with social support (*r* = 0.141, *P* < 0.001) and negatively related to frailty (*r* = − 0.147, *P* < 0.001). There was a significant negative correlation between social support and frailty (*r* = − 0.186, *P* < 0.001). Stratified by gender, in the male subsample, frailty was negatively correlated with socioeconomic status (*r* = − 0.108, *P* < 0.05) and social support (*r* = − 0.141, *P* < 0.01). But there was no significant association between socioeconomic status and social support (*r* = 0.100, *P* > 0.05). In the female subsample, socioeconomic status was positively related to social support (*r* = 0.159, *P* < 0.001) and negatively associated with frailty (*r* = − 0.129, *P* < 0.01). Social support was negatively related with frailty (*r* = − 0.211, *P* < 0.001).

**Table 2:** Correlation between key variables

Variables	1.Socioeconomic status	2.Social support	3.Frailty
Total sample (*n* = 936)			
1.Socioeconomic status	1.000		
2.Social support	0.141^***^	1.000	
3.Frailty	− 0.147^***^	− 0.186^***^	1.000
Male subsample (*n* = 359)			
1.Socioeconomic status	1.000		
2.Social support	0.100	1.000	
3.Frailty	− 0.108^*^	− 0.141^**^	1.000
Female subsample (*n* = 577)			
1.Socioeconomic status	1.000		
2.Social support	0.159^***^	1.000	
3.Frailty	− 0.129^**^	− 0.211^***^	1.000

^***^P − value < 0.001,^**^P − value < 0.01, ^*^P − value < 0.05

### The mediating effect of social support

Table 3 displays the results of the mediation analysis for the total sample, male subsample and female subsample. In the total sample, Model 1 showed that higher socioeconomic status was significantly associated with lower frailty scores (*β* = − 0.099, *P* < 0.01). Model 2 showed that higher socioeconomic status was significantly related to higher levels of social support (*β* = 0.082, *P* < 0.01). When social support was included in Model 3 as a mediator, there was still a significant relationship between higher socioeconomic status and lower frailty scores (*β* = − 0.088, *P* < 0.01). Thus, social support partially mediated the link between socioeconomic status and frailty. We reached the same conclusion in the female subsample instead of the male subsample.

**Table 3:** Association of socioeconomic status and social support with frailty

Variables	Model 1 (Frailty)			Model 2 (Social support)				Model 3 (Frailty)	
	β	t		β		t		β	t
Total sample (*n* = 936)									
Gender	0.128	3.930^***^		0.029		0.985		0.132	4.091^***^
Age	0.103	3.066^**^		− 0.036		− 1.180		0.098	2.932^**^
Marital status	− 0.050	− 1.502		0.427		14.121^***^		0.012	0.328
Chronic disease	0.116	3.639^***^		− 0.070		− 2.391^*^		0.106	3.341^***^
BMI	0.015	0.477		0.091		3.068^**^		0.028	0.884
Sleep quality	0.073	2.313^*^		0.004		0.143		0.074	2.351^*^
Socioeconomic status	− 0.099	− 2.992^**^		0.082		2.686^**^		− 0.088	− 2.650^**^
Social support								− 0.144	− 4.047^***^
*R* ^2^	0.074				0.223			0.090	
*F*	10.586^***^				38.009^***^			11.463^***^	
Male subsample (*n* = 359)									
Age	0.077	1.424		− 0.014		− 0.297		0.075	1.401
Marital status	− 0.043	− 0.784		0.464		9.618^***^		0.005	0.078
Chronic disease	0.065	1.193		− 0.107		− 2.210^*^		0.054	0.988
BMI	− 0.044	− 0.811		0.131		2.759^**^		− 0.030	− 0.557
Sleep quality	0.046	0.879		− 0.083		− 1.794		0.038	0.715
Socioeconomic status	− 0.093	− 1.738		0.060		1.277		− 0.087	− 1.623
Social support								− 0.103	− 1.705
*R* ^2^	0.027			0.243				0.035	
*F*	1.655			18.874^***^				1.841	
Female subsample (*n* = 577)									
Age	0.120	2.689^**^		− 0.063		− 1.544		0.109	2.464^*^
Marital status	− 0.037	− 0.868		0.415		10.635^***^		0.037	0.795
Chronic disease	0.155	3.800^***^		− 0.058		− 1.555		0.145	3.584^***^
BMI	0.041	0.998		0.062		1.655		0.052	1.279
Sleep quality	0.094	2.308^*^		0.072		1.927		0.107	2.646^**^
Socioeconomic status	− 0.101	− 2.383^*^		0.088		2.293^*^		− 0.085	− 2.027^*^
Social support								− 0.178	− 3.926^***^
*R* ^2^	0.073				0.231			0.097	
*F*	7.472^***^				28.530^***^			8.769^***^	

^***^P − value < 0.001,^**^P − value < 0.01, ^*^P − value < 0.05

**Fig. 1:**
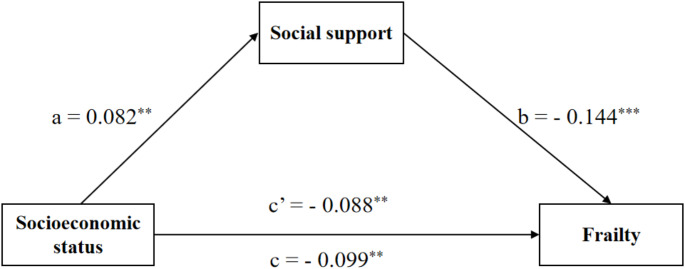
Path diagram of the association between socioeconomic status and frailty with social support as a mediator in the total sample. *Notes*: ^***^P-value < 0.001, ^**^P-value < 0.01. Models control for gender, age, marital status, chronic disease, BMI and sleep quality

**Fig. 2:**
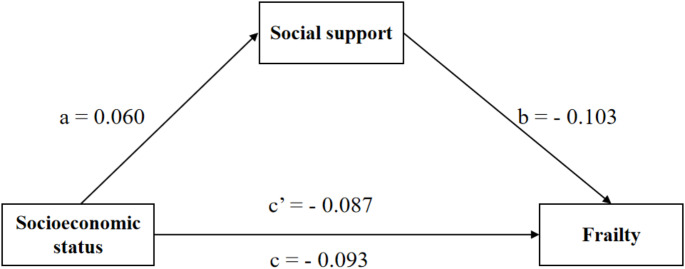
Path diagram of the association between socioeconomic status and frailty with social support as a mediator in the male subsample. *Notes*: All paths are not significant. Models control for age, marital status, chronic disease, BMI and sleep quality

**Fig. 3:**
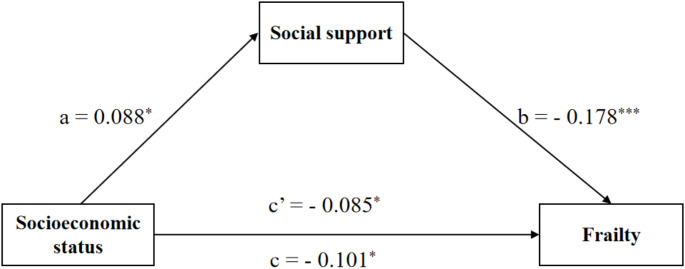
Path diagram of the association between socioeconomic status and frailty with social support as a mediator in the female subsample. *Notes*: ^***^P-value < 0.001, ^*^P-value < 0.05. Models control for age, marital status, chronic disease, BMI and sleep quality

Table 4 presents that, in the total sample, the total effect of socioeconomic status on frailty was − 0.099 (95% *CI*: − 0.153, − 0.032), and the indirect effect through social support was − 0.011 (95% *CI*: − 0.023, − 0.003), accounting for 11.11% of the total effect. In the female subsample, the total effect of socioeconomic status on frailty was − 0.101 (95% *CI*: − 0.181, − 0.018), and the indirect effect through social support was − 0.016 (95% *CI*: − 0.033, − 0.002), accounting for 15.84% of the total effect. Nonetheless, we did not find significant total, direct and indirect effects of socioeconomic status on frailty among the male subsample. Figures 1, 2 and 3 present the mediation pathway models for the total sample, male subsample and female subsample, respectively.

**Table 4:** Test of the mediating effect of social support

	Effect	SE	95% CI			Mediation (%)
			Lower	Upper		
Total sample (*n* = 936)						
Total effect	− 0.099	0.031	− 0.153	− 0.032		
Direct effect	− 0.088	0.031	− 0.142	− 0.021		
Indirect effect	− 0.011	0.005	− 0.023	− 0.003		11.11
Male subsample (*n* = 359)						
Total effect	− 0.093	0.046	− 0.169	0.010		
Direct effect	− 0.087	0.046	− 0.163	0.016		
Indirect effect	− 0.006	0.006	− 0.021	0.004		6.45
Female subsample (*n* = 577)						
Total effect	− 0.101	0.042	− 0.181		− 0.018	
Direct effect	− 0.085	0.041	− 0.165		− 0.003	
Indirect effect	− 0.016	0.008	− 0.033		− 0.002	15.84

## Discussion

The present study examined the relationship between socioeconomic status and frailty among rural older adults and for the first time explored the mediating role of social support in this relationship, as well as gender differences in the association among socioeconomic status, social support, and frailty. Our results revealed that there was a significant negative relationship between socioeconomic status and frailty among rural older adults. That said, those with low socioeconomic status were at higher risk of frailty. Meanwhile, we found that the effect of socioeconomic status on frailty was partially mediated through social support. The mediating effect of social support accounted for 11.11% of the total effect of socioeconomic status on frailty. An intriguing finding is that there were gender differences in the above results. Specifically, we discovered that, only in females, the relationship between socioeconomic status and frailty held and the mediating effect of social support was also significant.

Our finding that increased socioeconomic status was associated with decreased symptoms of frailty after adjusting for all covariates was in line with previous studies [53]. Individuals with higher socioeconomic status show higher compliance with interventions related to health promotion [54]. They tend to pay more attention to their own health, have a higher willingness to enrich health knowledge through various channels, and urge themselves to develop good living habits. Likewise, groups with higher socioeconomic status may have greater access to medical services and be at an advantage in obtaining more specialized and complex medical services [55]. All of these factors could be effective in reducing the occurrence of frailty or delaying its aggravation. Prior research has confirmed that socioeconomic status may also be linked to frailty through diet. Low socioeconomic status groups are more restricted in dietary choices and more difficult to achieve balanced nutrition, making them vulnerable to malnutrition [56]. It may also increase the level of inflammation in the blood [57, 58], thereby reducing bone mineral density and even leading to osteoporosis [59]. There is recent evidence that individuals with low socioeconomic status are more likely to drink alcohol [60], which can increase the risk of obesity [61, 62]. As we all know, obesity has been proven to be an independent risk factor for certain diseases [63–65]. It can also induce the pro-inflammatory state through the release of adipokines, accelerating muscle loss and altering the composition and quality of muscle [66], and ultimately promoting the onset and progress of frailty.

This study also analyzed the role of social support in the relationship between socioeconomic status and frailty. Our results suggested that social support could partially mediate the effect of socioeconomic status on frailty. This finding is new and additional evidence for existing related research. On one hand, groups with high socioeconomic status may receive more social support. This is because they are usually more connected to the outside world, have wider social networks, and thus can receive more social support from richer sources [67]. Moreover, the social networks of those are more stable [68]. Socioeconomic status can also influence social support through life events. This means that people with low socioeconomic status may experience more negative life events, leading to disrupted social relationships and reduced social support [69]. On the other hand, low levels of social support may increase the risk of frailty. Social support is an important driving force for health promotion behaviors for older adults. The material and spiritual help provided by members of the social network is conducive to reducing the burden of older adults, improving their ability to access health information and increasing their confidence in self-health management [70], thereby better improving their health level. In addition, adequate social support plays an important role in effectively relieving stress [71]. People with low levels of social support may feel more pressure and loneliness, have difficulty in resolving adverse emotions and be prone to a series of psychiatric problems or illnesses [72, 73], as well as potentially affecting sleep quality [74, 75], which can increases the risk of frailty. Additionally, previous research has indicated that low levels of social support and frailty could also be linked due to inflammation [76].

Our study also explored gender differences in the relationship among socioeconomic status, social support and frailty. The results showed that when stratified by gender, the relationship between socioeconomic status and frailty and the mediating effect of social support were significant only in the female subsample. There may be several reasons: Firstly, while the sensitivity to socioeconomic status is not inherently gender-specific, prior results have been found that females may be more sensitive to perceptions of socioeconomic status than males [77]. For example, females tend to show stronger emotional reactions when faced with changes in their economic situation [78], increasing the risk of mental and physical health problems. Secondly, the risk of frailty varies by gender [79, 80], which may imply the results of the present study. Previous studies have shown that females have higher inflammatory levels and greater inflammatory activities, and that gradually declining estrogen level with age is significantly associated with adverse events such as decreased bone density and increased risk of cardiovascular disease, making them more susceptible to frailty [81]. Finally, compared with males, females are better at emotional expression and show more comfort in socializing [82], which makes it easier for them to establish and maintain close and lasting relationships with others, thus possibly gaining more social support. As an important protective factor against frailty, the higher level of social support has a significant effect on reducing susceptibility to frailty [35].

Of course, these causes do not ensure that the relationship among socioeconomic status, social support and frailty is not statistically significant in the male population. Our findings may be influenced by several factors. Previous studies have shown differences in the types of social support exchanged by males and females. Specifically, compared with males, females exchanged more emotional support with others in terms of receiving and giving [83]. Meanwhile, females tend to have more same-sex friends [84], which is beneficial to their higher levels of emotional social support. There is recent evidence that emotional social support is more strongly associated with frailty than instrumental social support among older adults [85]. This may serve as a substrate for the phenomenon that the current mediation model is not significant in the male subsample. Secondly, our results may also be affected by the insufficient sample size included, as a small sample size may reduce statistical power. In addition, it cannot be ignored that the measurement instrument used in this study may also have measurement invariance limitations concerning the gender dimension.

Our results have some implications for the prevention and intervention of frailty among older adults. To begin with, the government and other institutions should provide more policy support and social welfare for older people with low socioeconomic status to meet their basic needs for livelihood and health care services. Secondly, it is necessary to actively build social platforms and organize various community activities regularly to help older adults broaden their interpersonal network and maintain optimism. Besides, older adults should be encouraged to exercise consciously to enhance physical fitness, get rid of improper behaviors and cultivate healthy living habits, so as to prevent the occurrence of frailty. At last, public health departments should focus on older adults with low socioeconomic status and lack of social support. It is important to strengthen monitoring and screening for frailty and implement comprehensive interventions as early as possible for those who are already experiencing frailty or prefrailty to prevent the exacerbation of symptoms and facilitate the reversal to health.

This study also has several limitations. Firstly, as a cross-sectional study, it was not the ideal form for determining causality between variables. Secondly, the proportions of mediating effects in the total and female samples reflected the fact that other potential mechanisms may need further to be explored in the relationship between socioeconomic status and frailty. Thirdly, some of the data, such as frailty, social support and sleep quality, were obtained by self-report using well-established scales, which was inevitably subject to reporting bias and recall bias. Fourthly, the non-significant findings in the male subsample resulting from the present study were not completely established. Affected by some factors such as sample size and measurement limitations, our research is rather shallow in exploring the gender differences in the relationship among the three key variables, which suggests that more in-depth analyses are necessary in the future. Furthermore, future research should continue to explore other factors influencing frailty in both males and females, and use social support measures that capture gender-specific differences in the types and dynamics of support provided or received by both sexes. Finally, participants in this study were only from rural areas in an eastern province of China. In order to improve the generalizability of our findings, the scope and population of the study should be expanded in the future.

## Conclusion

In conclusion, this study demonstrated that socioeconomic status was significantly associated with frailty among rural older adults, and that social support mediated this relationship. In addition, we found that there were gender differences in the relationship among socioeconomic status, social support and frailty. Specifically, the association between socioeconomic status and frailty and the mediating role of social support were statistically significant in the female subsample but not in the male subsample. These findings highlighted the importance of socioeconomic status and social support in improving the frailty of rural older adults, especially females. Additional studies need to be conducted in the future to explore other underlying pathways and temporal sequence between socioeconomic status and frailty.

## Data Availability

The datasets used and/or analyzed during the current study are available from corresponding authors on reasonable request.
